# Identification of Survival-Associated Gene Signature in Lung Cancer Coexisting With COPD

**DOI:** 10.3389/fonc.2021.600243

**Published:** 2021-03-09

**Authors:** Ti-wei Miao, Long-yi Du, Wei Xiao, Bing Mao, Yan Wang, Juan-juan Fu

**Affiliations:** ^1^Respiratory Group, Department of Integrated Traditional Chinese and Western Medicine, West China Hospital, Sichuan University, Chengdu, China; ^2^Research Core Facility, West China Hospital, Sichuan University, Chengdu, China

**Keywords:** COPD, lung cancer, survival, meta-analysis, DEGs (Differentially Expressed Genes)

## Abstract

**Background:** Chronic obstructive pulmonary disease (COPD) and lung cancer often coexist, which is associated with a worse prognosis. Thousands of biomarkers related to the survival of lung cancer have been investigated. However, those which can predict the survival of lung cancer coexisting with COPD are currently lacking. The present study aimed to identify novel gene signatures to predict the survival of patients with lung cancer coexisting COPD.

**Method:** RNA-sequence data of lung cancer and control accompanying with matched clinical information were retrieved from the Cancer Genome Atlas (TCGA). Differently expressed genes (DEGs) associated with lung cancer coexisting COPD were screened. Gene Ontology (GO) and Kyoto Encyclopedia of Genes and Genomes (KEGG) were performed. Univariate and multivariate Cox regression analyses were applied to identify survival-associated DEGs and to construct survival-associated gene signature. Kaplan-Meier survival analysis and calibration plots of the nomogram were performed to test the predictive accuracy of the gene signature. qPCR was performed to validate the genes in the prognostic signature.

**Results:** Sequence data from 70 patients with lung cancer coexisting COPD, 127 with lung cancer alone and 108 control tissues were included for analysis. A total of 2424 DEGs were identified when comparing lung cancer coexisting COPD with controls. The biological process was primarily associated with DNA-binding transcription activator activity, peptidase inhibitor activity, endopeptidase inhibitor activity, et al. KEGG pathways were mainly enriched in neuroactive ligand-receptor interaction, cell cycle, and *Staphylococcus aureus* infection. A survival-associated gene signature consisting of *CEACAM5, RASAL1, CSTL1, CNGB1*, and *SLC4A3* was identified and represented as risk score. The high-risk score group had significantly worse survival than the low-risk score group (*P* < 0.001). Areas under receiver operating characteristic curves were 0.943, 0.773, 0.888 for predicting overall survival at 1-, 3-, and 5-year, respectively. The risk score was an independent predictor of survival, independent of clinical factors. High conformity of the actual survival and the nomogram–predicted probability of survival by applying the risk score. Upregulation of the five genes in patients with lung cancer coexisting COPD were confirmed by qPCR in an independent cohort.

**Conclusion:** Our study constructed and validated a novel prognostic gene signature for predicting survival of patient with lung cancer coexisting COPD, which may contribute to the clinical treatment decisions.

## Introduction

Chronic obstructive pulmonary disease (COPD) and lung cancer are leading cause of morbidity and mortality worldwide. The overall prevalence of spirometry-defined COPD is 8.6% among the general population aged 20 years or older in China (1). It is currently the third leading cause of death worldwide (2). Lung cancer is the world's leading cause of cancer death with a low 5-year overall survival (OS) rate (3, 4) and most patients are diagnosed at an advanced incurable stage (5). The incidence of these two diseases have been increasing in recent years and this trend is estimated to continue. It has been found that 50–80% of patients with lung cancer coexist with COPD, and the prevalence of lung cancer is significantly higher in patients with COPD than those without COPD (6, 7), and the presence of emphysema in COPD predicts a higher lung cancer risk adjusted for smoking status (8, 9).

Importantly, patients with lung cancer coexisting COPD had a worse prognosis compared to patients with lung cancer alone. For non-small cell lung cancer (NSCLC), the median survival time was shorter in patients with COPD compared to those without COPD (10). In terms of prognosis of surgical resection of lung cancer, OS, and disease-free survival in the COPD group were significantly worse than the non-COPD group (11, 12). Patients with COPD undergoing lung cancer surgery were at higher risk of postoperative complications than patients with normal respiratory function (13). In addition, patients with advanced NSCLC coexisting COPD complained of more symptoms, decreased lung function, lower quality of life, and shorter overall median survival (14).

In recent years, a variety of biomarkers have been reported which are associated with OS of lung cancer, such as S100A4 (15), lymphocyte to C-reactive protein ratio (16), mini-chromosome maintenance complex component 7 (*MCM7*), and cyclin E2 (*CCNE2*) (17), LY6/PLAUR domain containing 3 (*LYPD3*) (18). However, some studies have found that gene signatures comprising a panel of genes have better predictive capability on prognosis and survival than a single gene or biomarker. For instance, autophagy-related gene signatures and alternative splicing signatures can effectively predict OS of patients with lung cancer (19–21). N^6^-methyladenosine-related gene signatures, glycolysis-related gene signatures were associated with prognosis of patients with lung adenocarcinoma (22–24). The genetic signature closely related to the tumor immune microenvironment could predict the prognosis of patients with NSCLC (25). However, our knowledge on gene signatures that predict the prognosis of patients with lung cancer coexisting COPD is vacant but deserves investigation given the increasing coexistence of both diseases and the poor prognosis.

In the present study, we aimed to explore gene signature for predicting survival of patients with lung cancer coexisting COPD by accessing RNA-sequence data from the Cancer Genome Atlas (TCGA) database and using bioinformatic analyses.

## Materials and Methods

### Data Acquisition

RNA-sequence data of lung cancer and control tissues accompanying with matched clinical information were obtained from the TCGA (https://tcga-data.nci.nih.gov/tcga/). TCGA is a landmark cancer genomics program containing over 20,000 primary cancer and matched normal samples spanning 33 cancer types. Patients with lung cancer with FEV_1_ (forced exhalation volume in 1 s)/FVC (forced vital capacity) < 0.7 for spirometry were defined as lung cancer coexisting COPD and were included in current study for further analysis. Data processing was performed by R language (Version 4.0.2) and Perl (Version 5.30.2.1).

### Identification of Differentially Expressed Genes (DEGs) and Functional Enrichment Analysis

The “limma package” in R software was applied to screen DEGs for lung cancer coexisting with COPD compared to controls. Genes with |log FC| ≥ 2 in expression level and adjusted *P* < 0.05 were considered as statistically significant DEGs when two groups were compared. The “ggplot2 package” in R software was used to perform the volcano plots of all genes. Enrichment analysis of Gene Ontology (GO) and Kyoto Encyclopedia of Genes and Genomes (KEGG) pathway was performed using DEGs in R language. GO mainly depicts biological process in current study. *P* < 0.05 was regarded as an accepted threshold criterion.

### Identification of Survival-Associated Gene Signature

Univariate Cox regression analysis was conducted to identify candidate genes that were significantly correlated with OS with *P* < 0.05. The genes were further selected by multivariate Cox regression analysis. The survival-associated gene signatures were calculated as a risk score according to a linear combination of the expression values of selected gene multiplied by a regression coefficient (β) accessed from the multivariate Cox proportional hazards regression model for each gene. The formula was as follow: risk score = expression of gene 1 × β1 gene 1 + expression of gene 2 × β2 gene 2 +… expression of gene n × βn gene n. All patients were divided into low- or high-risk groups according to the median risk score for included patients.

### Predictive Accuracy of the Survival-Associated Gene Signature

OS of the patients with high and low risk scores were compared by Kaplan-Meier survival analysis in patients with lung cancer coexisting COPD and those with lung cancer alone. Receiver operating characteristic (ROC) analysis was performed to assess the predictive accuracy of gene signature represented as risk score. Univariate Cox hazard analyses were performed for risk score and clinical characteristics, and multivariate model was constructed adjusting for significant clinical characteristics with age and sex forced in the model.

### Nomogram Predicting Model

A nomogram plot was applied to forecast the likelihood of OS at 1-, 3-, and 5- year using the “rms package” in R language. The conformity of nomogram–predicted probability of OS and actual OS was compared by calibration plots of the nomogram for 1-, 3-, and 5- year survival.

### RNA Extraction and qPCR Validation

Lung tissue specimens were obtained from patients who underwent lobectomy or pneumonectomy for lung cancer in West China Hospital of Sichuan University. After surgical resection, lung tissues were immediately frozen to −80°C. Histologically normal tissues adjacent to the tumor were used as controls. Patients with lung function of FEV_1_/FVC<0.7 were defined as lung cancer coexisting COPD. Total RNA was extracted from lung tissues (22 controls, 12 lung cancer, and 22 lung cancer coexisting with COPD) according to the manufacturer's protocol using the E.Z.N.A. HP Total RNA Kit (OMEGA, USA). PrimeScript™ RT reagent Kit with gDNA Eraser (Takara, Japan) was applied to synthesize cDNA following the manufacturer's instructions. qPCR was performed in triplicate by using Iq™ SYBR Green Supermix (BIO-RAD, USA) according to the manufacturer's protocol. Relative expression level of gene was normalized via the GAPDH Ct value (endogenous reference), applying a 2^−ΔΔCt^ relative quantification method. The qPCR primers were as follow:

*CEACAM5*-forward: 5′- CCGTGGAGGACAAGGAT-3′

*CEACAM5*-reverse: 5′- GGGTTCTGGGTTTCACATT-3′

*RASAL1*-forward: 5′- TGGCACATCTGACCCAT-3′

*RASAL1*-reverse: 5′- CTCCAGCACTTCATCCCA-3′

*CNGB1*-forward: 5′- CACGGCCAGCACAAATA-3′

*CNGB1*-reverse: 5′- TTGGGGCTCTCCTCATC-3′

*CSTL1*-forward: 5′- CCCCAGGAAAGCCTATGT-3′

*CSTL1*-reverse: 5′- TCCGATCCCCATGTCTAC-3′

*SLC4A3*-forward: 5′- AGGCTCGTCGCATCATC-3′

*SLC4A3*-reverse: 5′- CGCTTATCGGGAGAGGTC-3′

*GAPDH*-forward: 5′- TGCACCACCAACTGCTTAGC-3′

*GAPDH*-reverse: 5′- GGCATGGACTGTGGTCATGAG-3′.

### Statistical Analysis

Statistical analyses were performed with SPSS version 22 (IBM Corporation, USA). Levels of mRNA expression were expressed as median (interquartile range). Comparisons were made between two groups in terms of continuous data using Mann-Whitney *U*-test, and *P* < 0.05 was considered statistically significant. Statistical significance among multiple groups was tested by Kruskal-Wallis test, and an adjusted *P* < 0.017 was considered to be statistically significant to compare differences between three groups.

## Results

### Data Acquisition

**Figure 1:**
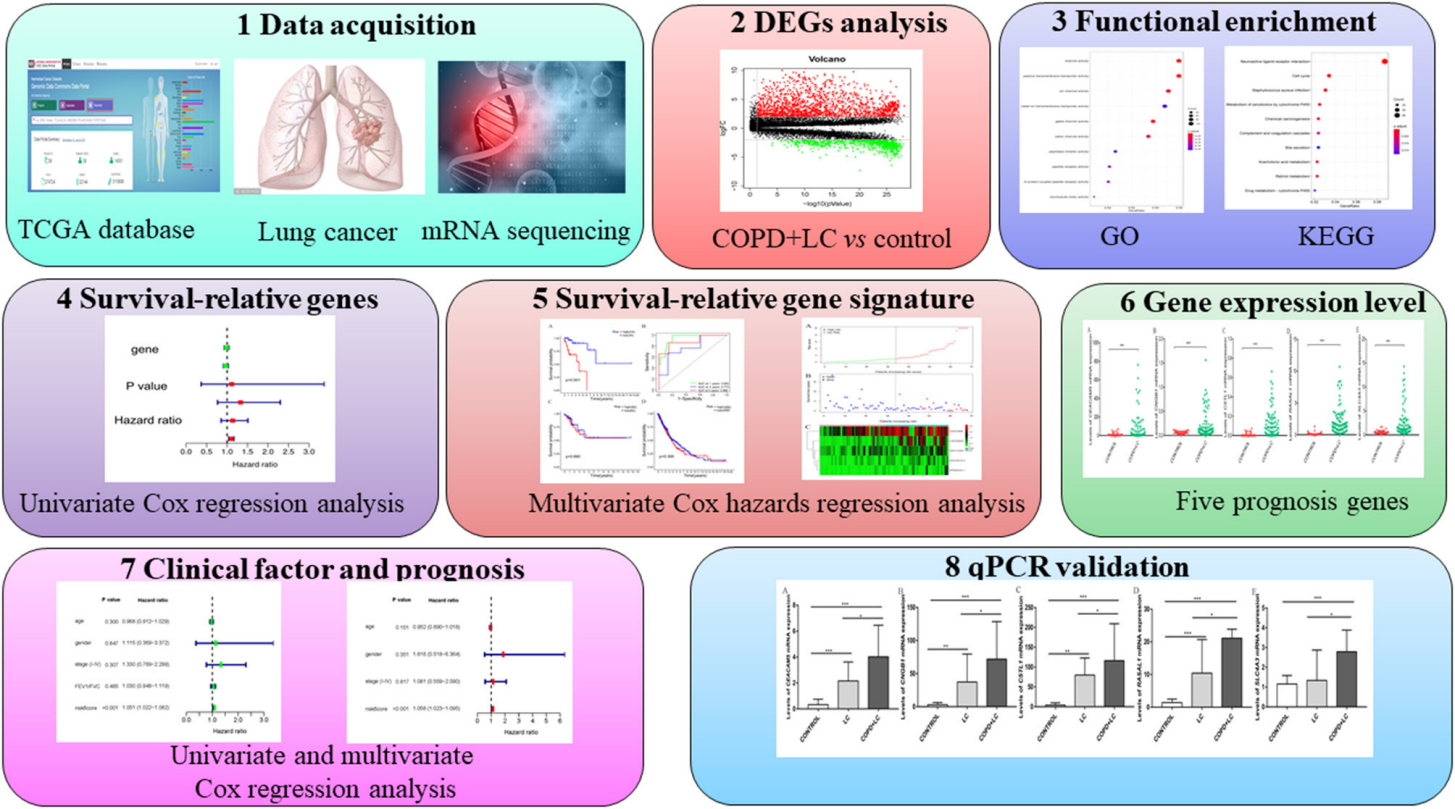
Study flow chart and main findings. *P < 0.05; **P < 0.01; ***P < 0.001.

The whole study flow is shown in Figure 1. RNA-sequence data of lung tissues samples from 1,145 patients (lung cancer = 1,037, healthy control = 108) were retrieved. Clinical information including the gender, age, tumor stage, pulmonary function, survival time, and survival status were available for 1,027 patients with lung cancer. Data of lung function were available in 197 patients with lung cancer, in which 70 patients were defined as lung cancer coexisting with COPD. The baseline characteristics of patients with lung cancer coexisting COPD were listed in Table 1.

**Table 1:** Clinical features of patients with COPD coexisting with lung cancer (*n* = 70) from TCGA.

**Clinical characteristic**	***N***	**%**
**Age (years)**		
>65	40	57.14
≤65	30	42.86
**Gender**		
Male	38	54.28
Female	32	45.72
**T classification**		
T1-2	60	85.71
T3-4	10	14.29
**N classification**		
N0	49	70.00
N1-3	21	30.00
**M classification**		
M0	39	55.72
M1-Mx	31	44.18
**UICC stage**		
Stage I-II	56	80.11
Stage III–IV	12	17.14
**GOLD stage**		
Stage I-II	53	75.71
Stage III–IV	10	14.28

### DEGs and Functional Enrichment Analysis

**Figure 2:**
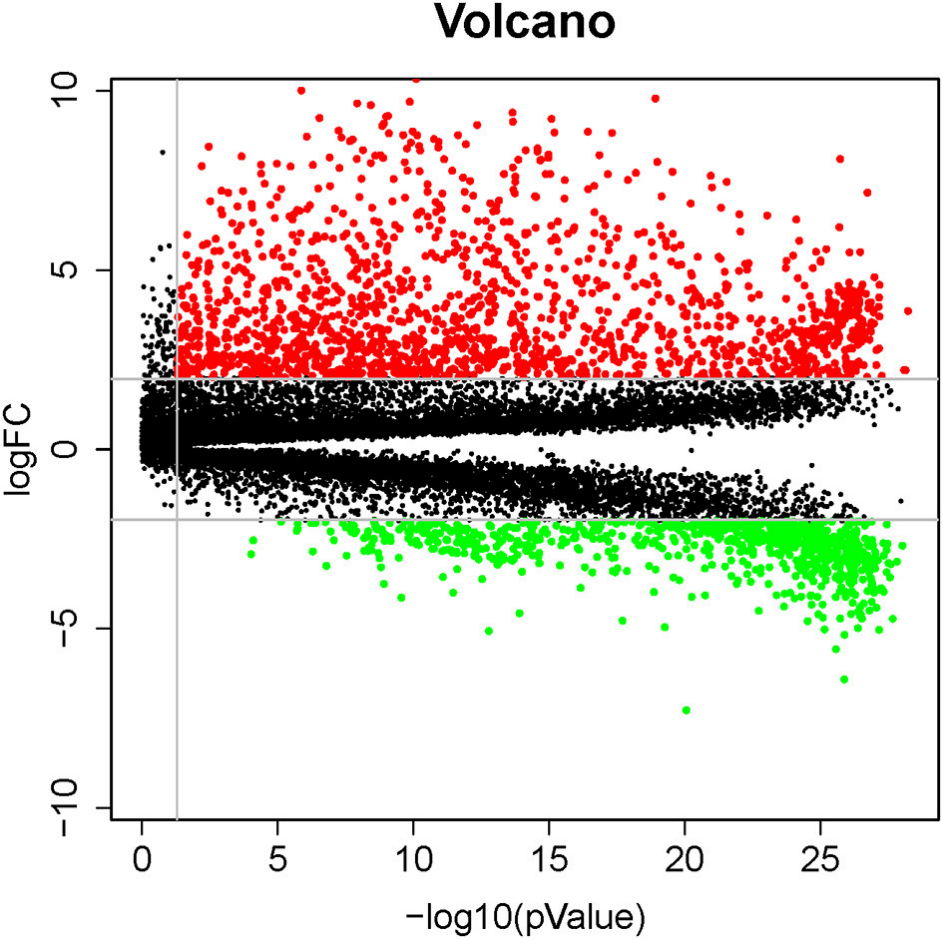
Volcano plots of differentially expressed genes involved in patients with lung cancer coexisting COPD vs. healthy controls. Red and green dots denote up-regulated and down-regulated genes, respectively.

**Figure 3:**
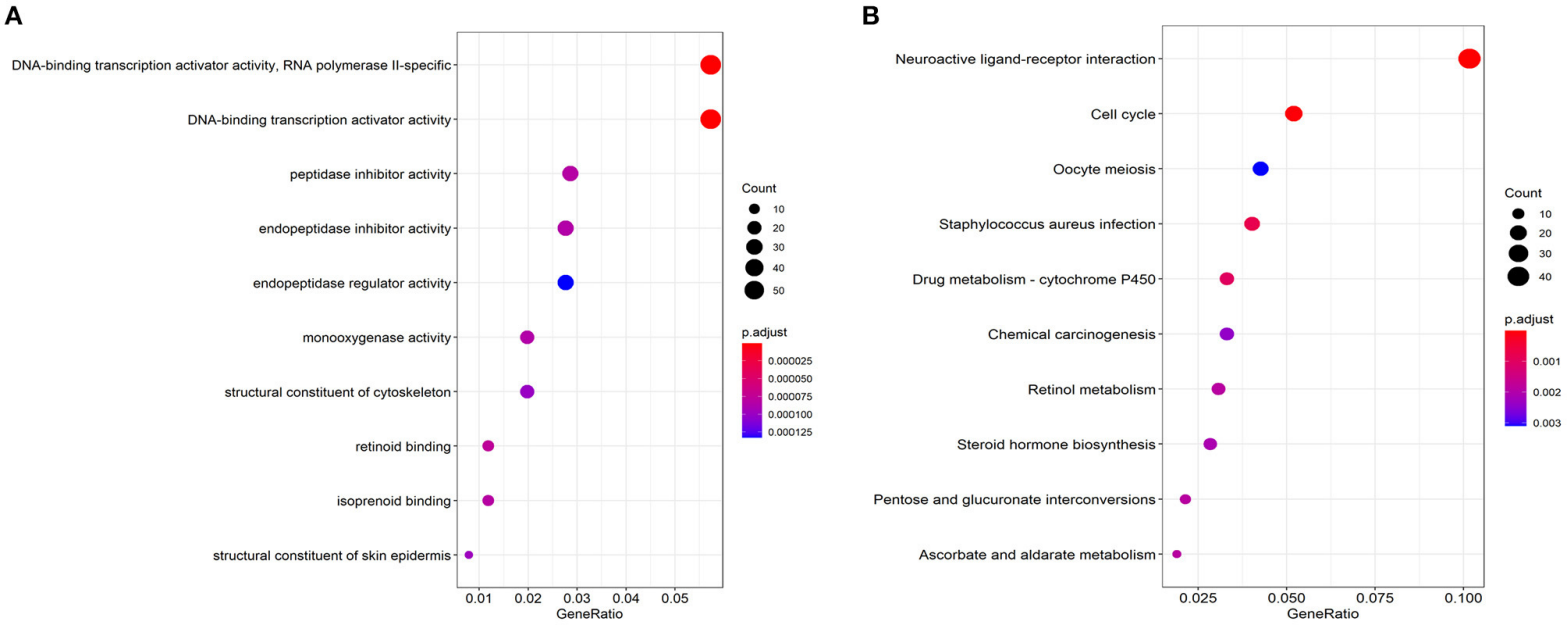
Top 10 significant enriched biological process **(A)** and KEGG pathways **(B)** based on DEGs related to lung cancer coexisting COPD.

There were 2424 DEGs (1,596 up-regulated and 828 down-regulated genes) when comparing lung cancer coexisting COPD to the controls (Figure 2). Functional enrichment analysis was performed using the 2424 DEGs. The biological process was primarily associated with DNA-binding transcription activator activity, peptidase inhibitor activity, endopeptidase inhibitor activity, et al. The top 10 biological process were listed in Figure 3A. The top 10 pathways were listed in Figure 3B, which were mainly enriched in the neuroactive ligand-receptor interaction, cell cycle, *Staphylococcus aureus* infection, et al.

### Identification of Survival-Associated Gene Signature

**Figure 4:**
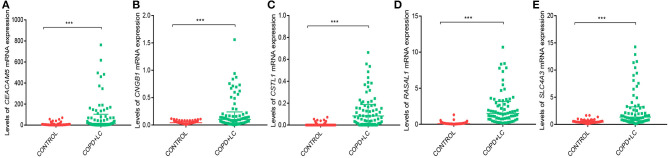
The levels of *CEACAM5* **(A)**, *CNGB1* **(B)**, *CSTL1* **(C)**, *RASAL1* **(D)**, *SLC4A3* **(E)** mRNA expression in lung cancer coexisting with COPD vs. healthy controls. ***P < 0.001.

Univariate Cox regression analysis was performed for the 2424 DEGs, which identified nine survival-associated genes (*CEACAM5, RASAL1, CSTL1, CNGB1, TUBB3, HIST1H2AI, PAH, SLC4A3, HIST1H4H*). Subsequently, five DEGs including *CEACAM5, RASAL1, CSTL1, CNGB1*, and *SLC4A3* were identified as independent predictor for OS by Multivariate Cox regression analysis. The five genes were significantly upregulated in lung cancer coexisting with COPD compared to controls (all *P* < 0.001) (Figure 4). The risk score formula was established based on a linear combination of the expression levels weighted with the regression coefficients derived from multivariate Cox regression analyses: Risk score = 0.1284 × expression of *CEACAM5* + 0.6739 × expression of *RASAL1* + 3.0882 × expression of *CSTL1* + 3.2026 × expression of *CNGB1* + 0.3574 × expression of *SLC4A3*. Patients were divided into low- or high-risk groups according to the median risk score at 0.78 (**Figure 6A**).

### Risk Score as an Independent Prognostic Indicator

**Figure 5:**
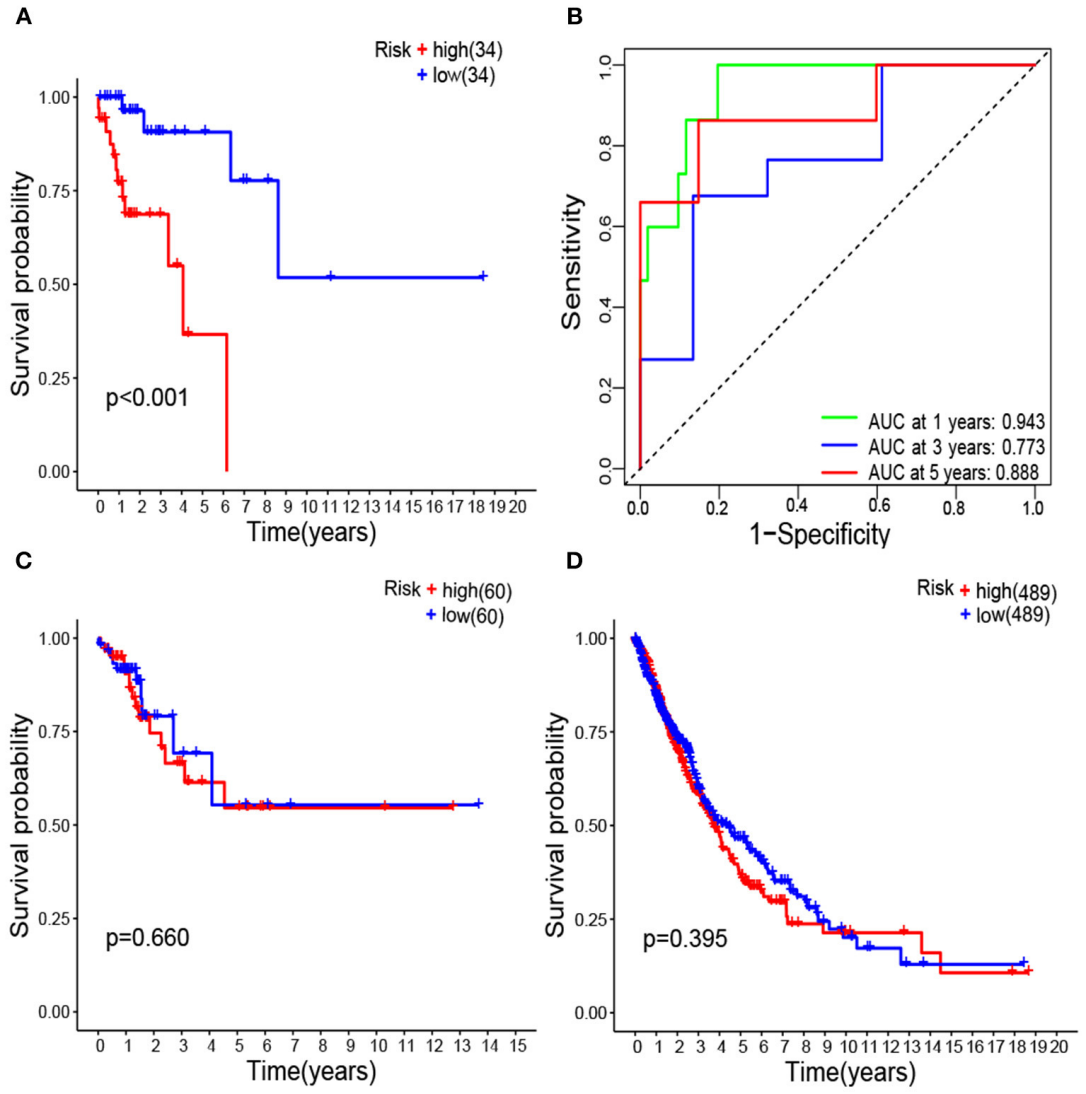
Kaplan–Meier survival analysis of survival-associated gene signature represented as risk score high vs. low in lung cancer coexisting COPD **(A)**; Receiver operating characteristic analysis of the survival-associated gene signature represented as risk score low in lung cancer coexisting COPD **(B)**; Kaplan–Meier survival analysis of survival-associated gene signature in lung cancer alone **(C)**; Kaplan–Meier survival analysis of survival-associated gene signature in all lung cancer regardless of lung function **(D)**.

The high-risk group had significant poor survival compared with the low-risk group (*P* < 0.001) in patients with lung cancer and coexisting COPD (Figure 5A).

**Figure 6:**
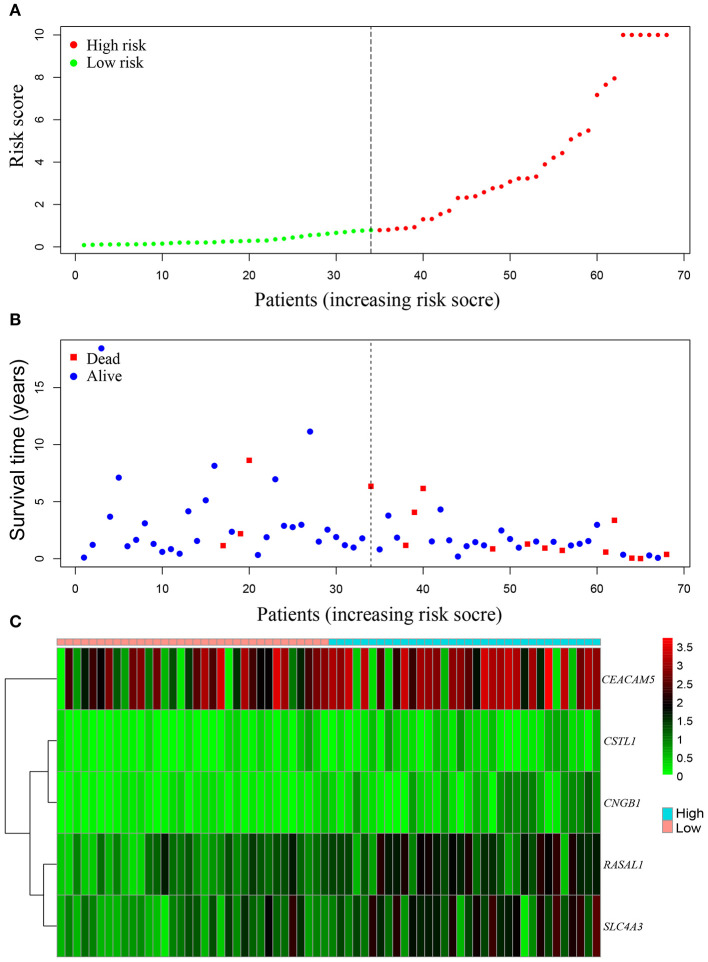
The survival-associated gene signature represented as risk score predicts the overall survival of lung cancer coexisting with COPD. Risk score distribution **(A)**. Survival status **(B)**. Heatmap of survival-associated gene expression profile in TCGA **(C)**.

**Figure 7:**
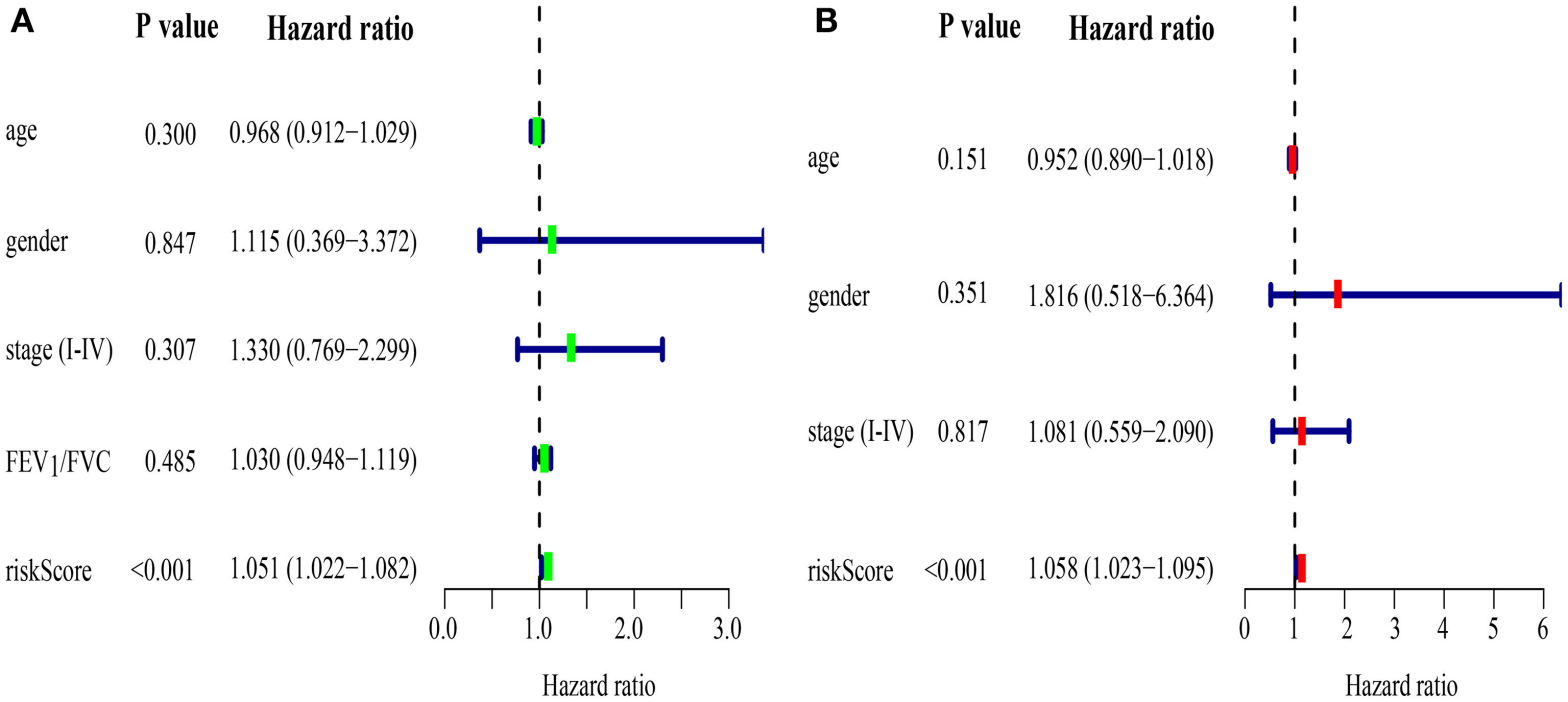
Prognostic model of survival in lung cancer coexisting COPD by univariate **(A)** and multivariate **(B)** Cox regression analyses.

Areas under ROC curves (AUC) were 0.943, 0.773, 0.888 for predicting OS at 1, 3, and 5-year, respectively (Figure 5B). Survival was neither different between the high and low risk groups in patients with lung cancer alone (Figure 5C), nor in all patients with lung cancer which did not take lung function or the coexistence of COPD into consideration (Figure 5D). The OS of each patient is shown in Figure 6B. A heatmap is shown to present the different expression profile of the five genes between the two risk groups (Figure 6C). The risk score was the only variables that was associated with OS in the univariate analysis (hazard ratio [HR]: 1.051; 95% confidence interval [CI]: 1.022–1.082; *P* < 0.001) and in the multivariate analysis (hazard ratio [HR]: 1.066; 95% confidence interval [CI]: 1.028–1.105; *P* < 0.001) (Figure 7).

### Nomogram Predicting Model

**Figure 8:**
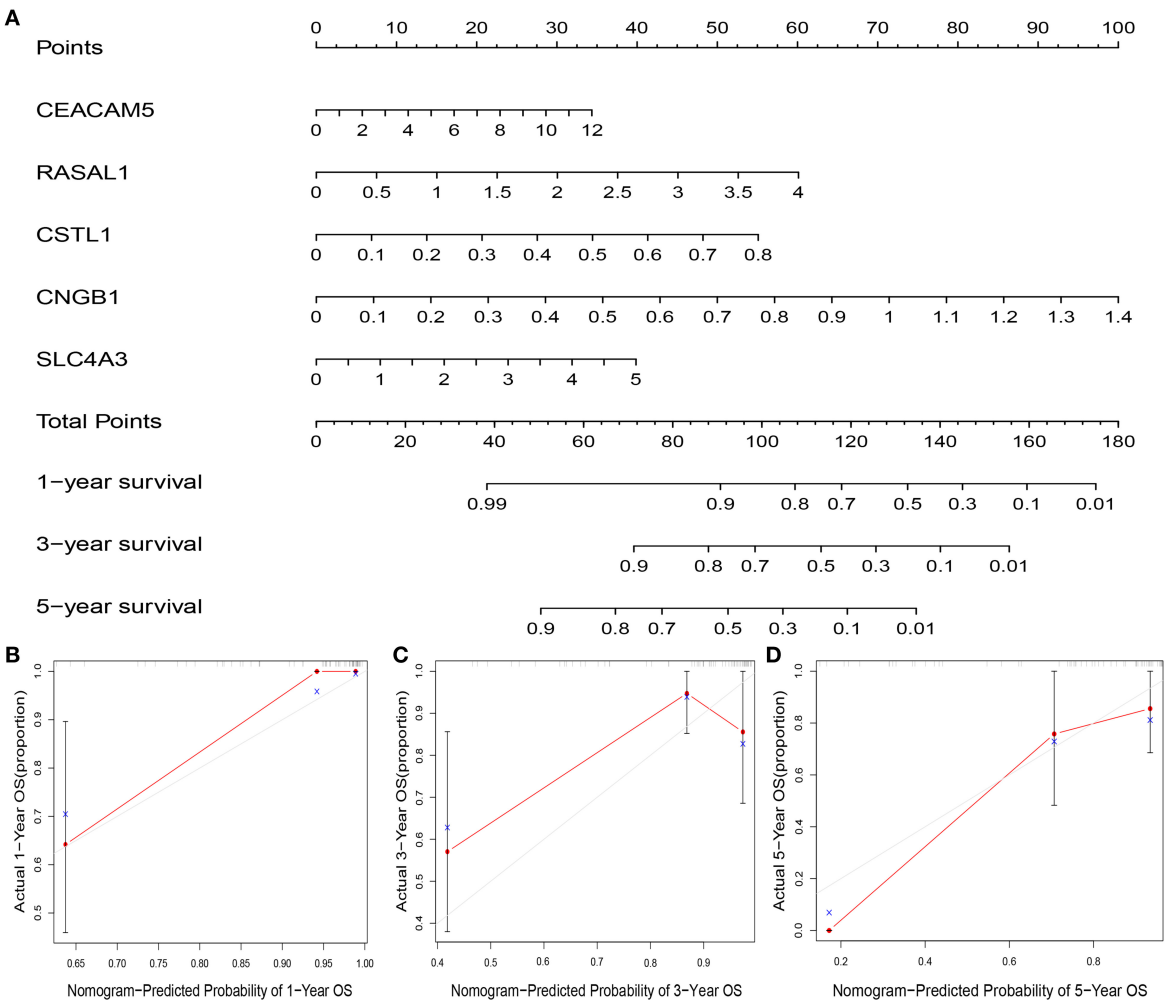
Nomogram and calibration plot of five genes and the risk score. Nomogram to predict overall survival at 1-, 3-, and 5-year **(A)**. Calibration plots of the nomogram to predict OS at 1-, 3-, and 5-year **(B–D)**. Nomogram-predicted overall survival is plotted on the x-axis, with actual overall survival on the y-axis. Gray lines represent the prefect calibration models in which the predicted probabilities (red lines) are identical to the actual probabilities of overall survival.

A nomogram plot was constructed using the five DEGs. This allowed us to calculate the estimated survival probabilities at 1, 3, and 5 -year by plotting a vertical line between the total point axis and each prognosis axis (Figure 8A). Calibration plots of the nomogram showed high conformity of nomogram–predicted probability and actual survival at 1, 3, 5-year (Figures 8B–D).

### qPCR Validation of the Expression Levels of the Five Prognostic Genes

**Figure 9:**
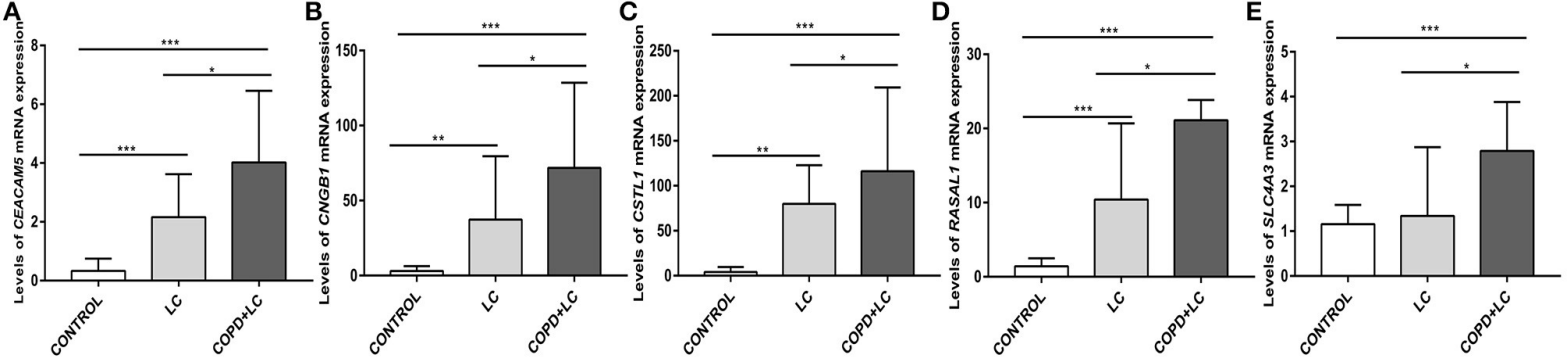
Validation of the levels of *CEACAM5* **(A)**, *CNGB1* **(B)**, *CSTL1* **(C)**, *RASAL1* **(D)**, *SLC4A3* **(E)** expression in lung tissues by qPCR. ^*^P < 0.05; **P < 0.01; ***P < 0.001.

The level of *CEACAM5* (*P* < 0.001), *CNGB1* (*P* = 0.001), *CSTL1* (*P* = 0.009), *RASAL1* (*P* < 0.001) expression were higher in lung tissues of patients with lung cancer alone than controls. The level of *CEACAM5* (*P* < 0.001), *CNGB1* (*P* < 0.001), *CSTL1* (*P* < 0.001), *RASAL1* (*P* < 0.001), *SLC4A3* (*P* < 0.001) expression were also increased in lung tissues of patients with lung cancer coexisting COPD compared to controls. In addition, the level of *CEACAM5* (*P* = 0.030), *CNGB1* (*P* = 0.042), *CSTL1* (*P* = 0.046), *RASAL1* (*P* = 0.039), *SLC4A3* (*P* = 0.035) expression were higher in patients with lung cancer coexisting COPD than that in patients with lung cancer alone (Figures 9A–E).

## Discussions

In current study, by accessing RNA-sequence data from TCGA, five survival-associated genes were identified by multivariate Cox regression analysis from the 2424 DEGs associated with the coexistence of lung cancer with COPD. A risk score was generated according to the five genes as a signature, which showed good predictive capacity on survival based on ROC and survival analysis, independent of stages of cancer. By qPCR validation in another cohort, the levels of five genes were significantly upregulated in lung tissues of patients with lung cancer coexisting COPD than lung cancer alone and controls.

The KEGGs in patients with lung cancer coexisting COPD was primarily associated with *S. aureus* infection, neuroactive ligand-receptor interaction, and cell cycle et al. Recent studies have revealed complex interaction between the microbiome and different diseases including lung cancer. Smoking, as a major risk factor for lung diseases, has been reported to alter microbial diversities and communities in the lower respiratory tract of both mice and human trials (26, 27). Staphylococcus aureus could influence the pathogenesis of COPD (28). LukS-PV produced by *S. aureus* can induce cell cycle arrest and apoptosis through p38/ERK MAPK signaling pathway in NSCLC cells (29), and lipoteichoic acids from *S. aureus* could stimulate proliferation of human NSCLC cells *in vitro* (30). Lung microbiota represented as *S. aureus* may be involved in the pathogenesis of both COPD and lung cancer. Further study is needed to elucidate how the microbiome engaged in the high prevalence of lung cancer in COPD. Recent studies have identified that smoking was involved in the activation of cell cycle in the development of COPD and COPD multimorbidities including the occurrence of lung cancer (31). These pathways warranted further study to reveal the mechanisms underlying the coexistence of both diseases.

CEACAM5 (Carcinoembryonic antigen-related cell adhesion molecules) is a family member of “carcinoembryonic antigen” (32). It is located in the stomach, tongue, esophagus, cervix, sweat glands, and prostate normally (33). CEACAM5 has also been found to be expressed in a wide variety of cancer (33). The level of CEACAM5 protein in serum of patients with NSCLC were significantly higher than those with benign pulmonary lesions (34, 35). Higher level of *CEACAM5* expression were associated with a worse prognosis in patients with NSCLC (36). *CEACAM5* expression level can estimate the presence of micrometastatic cells in lymph node with greater precision than current staging method used for assessing tumor recurrence risk (37). Importantly, *CEACAM5* has been found to be associated with the occurrence of COPD by WGCNA in a recent study (38). Our study firstly identified the upregulation of this gene in patients with lung cancer coexisting COPD and its role in the prediction on survival in this specific population, future study is warranted to reveal how it acts in COPD and in the development of lung cancer as well as disease prognosis.

RASAL1(RAS protein activator like 1) is a member of the RAS GTPase-activating protein (GAP) family (39), which participates in cellular proliferation and differentiation and is associated with pulmonary fibrosis (40), and cancer (41). A recent study showed that lncRNAPCAT29 inhibits pulmonary fibrosis via downregulating RASAL1/ERK1/2 signal pathway, which suppresses the expression levels of MMP2 and MMP9 in alveolar epithelial cells and pulmonary fibroblast cell differentiation (40). We found the upregulation of *RASAL1* expression in patients with lung cancer, and the level of gene expression was further elevated in those coexisting with COPD. The role of RASAL1 in COPD and lung cancer has not been investigated, which may relate to matrix metalloproteases involving in inflammation and cancer invasion and metastasis *CNGB1* (cyclic nucleotide gated channel subunit beta 1) encodes a cyclic nucleotide gated channel. It was first identified for its role in light activated cellular polarization in retinal photoreceptor cells. This ion channel related gene is also expressed in the small airway epithelium and it is a smoking-induced gene (42). CSTL1 is not prognostic for survival in lung cancer according to survival analysis using data in TCGA (https://www.proteinatlas.org/ENSG00000125823-CSTL1/pathology/lung+cancer). However, the above analysis including all patients with lung cancer which did not take lung function or the coexistence of COPD into consideration. As a part of our predictive panel which predicted survival in patients with lung cancer coexisting COPD, its function in lung cancer and COPD has not been addressed in literature but deserves further study. SLC4A3 protein acts as an anion exchange protein that has been associated with relapse time in lung cancer (43).

The risk score generated as a gene signature was associated with survival in patients with lung cancer coexisting COPD, however, it was not predictive for survival in patients with lung cancer alone and in all patients with lung cancer which did not take lung function or the coexistence of COPD into consideration. This indicates the predictive effect and value of this signature in this specific population with the coexistence of lung cancer and COPD. Previous study reported that the AUC of gene signatures for predicting prognosis of lung cancer were often no larger than 0.8 (19, 20, 23). In current study, the AUC of the gene signature represented as a risk score for predicting 1, 3, and 5-year survival were 0.943, 0.773, 0.888, respectively, showing a higher predictive value. The constructed nomogram plot showed a high conformity of nomogram–predicted probability of survival and actual survival in patients with lung cancer coexisting COPD. Notably, the risk score was an independent risk factor for survival in univariate and multivariate Cox regression analyses. Although the function and mechanisms of certain genes in lung cancer has not been clearly answered, these genes or as a prognosis predictive gene panel deserve further investigation (19, 20, 22, 24).

There are some highlights in current study. It is the first study that analyzed survival-associated genes and identified prognostic gene signature with a panel of five genes for lung cancer coexisting COPD by bioinformatic analysis, which may provide more accurate and reliable results than a single biomarker. We confirmed the predictive value of this signature by ROC analysis and multivariate regression model confounding for clinical characteristics. Moreover, the five genes was validated by qPCR in an independent cohort which showed higher expression levels of these genes in lung cancer coexisting COPD. The study also has certain limitations. We did not find independent cohort in other public databases e.g., the International Cancer Genome Consortium (ICGC) to validate the study findings. Although sample size for our validation cohort was small and we were unable to perform survival analysis due to that the expression levels of these genes were measured by qPCR which is different from RNA-sequence in the bioinformatic analysis, and that the newly collected samples were not matched with survival data, we did observe the upregulation of the five genes in lung cancer coexisting COPD. Larger cohort are needed to validate the study findings.

## Conclusion

A total of 2424 DEGs were identified in patients with lung cancer coexisting COPD by analyzing RNA-sequence data in TCGA. These DEGs were mainly associated with DNA-binding transcription activator activity, peptidase inhibitor activity, endopeptidase inhibitor activity, et al. KEGG pathways were mainly enriched in *S. aureus* infection, cell cycle and neuroactive ligand-receptor interaction. A panel of five survival-associated genes were identified, and a risk score generated from these five genes as a prognostic gene signature was shown to be an independent prognostic indicator for survival. High conformity of the actual survival and the nomogram–predicted probability of survival at 1, 3, 5 -year by using this risk score. The levels of five genes expression were upregulated in lung cancer coexisting COPD than lung cancer alone and controls in an independent validation cohort. This five-gene signature is promising for predicting survival of patients with lung cancer coexisting COPD deserving further investigation.

## Data Availability Statement

Publicly available datasets were analyzed in this study. This data can be found here: The Cancer Genome Atlas (https://portal.gdc.cancer.gov/).

## Author Contributions

T-wM and L-yD collected and analyzed data and drafted the manuscript. WX and YW analyzed data. BM and J-jF designed the experiment and revised the manuscript. All authors contributed to the article and approved the submitted version.

## Conflict of Interest

The authors declare that the research was conducted in the absence of any commercial or financial relationships that could be construed as a potential conflict of interest.
